# The Acceptability and Effectiveness of Web-Based Developmental Surveillance Programs: Rapid Review

**DOI:** 10.2196/16085

**Published:** 2020-04-23

**Authors:** Jess Baker, Jane Kohlhoff, Se-Inyenede Onobrakpor, Sue Woolfenden, Rebecca Smith, Constanze Knebel, Valsamma Eapen

**Affiliations:** 1 The University of New South Wales Liverpool Australia; 2 The University of New South Wales Carramarr Australia; 3 The Ingham Institute Liverpool Australia; 4 The University of New South Wales Sydney Australia; 5 South Eastern Sydney Local Health District Sydney Australia; 6 Westmead Children's Hospital Westmead Australia

**Keywords:** public health surveillance, mass screening, developmental disabilities, neurodevelopmental disorders, review literature as topic, health care disparities

## Abstract

**Background:**

Web-based developmental surveillance programs may be an innovative solution to improving the early detection of childhood developmental difficulties, especially within disadvantaged populations.

**Objective:**

This review aimed to identify the acceptability and effectiveness of web-based developmental surveillance programs for children aged 0 to 6 years.

**Methods:**

A total of 6 databases and gray literature were searched using a Preferred Reporting Items for Systematic Reviews and Meta-Analyses–informed protocol. Data extraction included variables related to health equity.

**Results:**

In total, 20 studies were identified. Most papers implemented web-based versions of the Modified Checklist for Autism in Toddlers, Revised with Follow-Up screener for autism spectrum disorder or Parent Evaluation of Developmental Status screeners for broad developmental delay. Caregivers and practitioners indicated a preference for web-based screeners, primarily for user-friendliness, improved follow-up accuracy, time, and training efficiencies.

**Conclusions:**

Although evidence is limited as to the necessity of web- versus face-to-face–based developmental screening, there are clear efficiencies in its use.

**Trial Registration:**

PROSPERO CRD42019127894; https://www.crd.york.ac.uk/prospero/display_record.php?RecordID=127894

## Introduction

### Background

Healthy early child development is an important predictor of emotional and physical well-being and school attainment, whereas (neuro)developmental difficulties such as communication or motor skills disorders and autism spectrum disorders (ASD) can hinder the fulfillment of optimal health and schooling [1]. The earlier such difficulties can be detected as an instigator of timely intervention, the better the outcome [2-6]. However, many developmental difficulties are not detected until school age [7]. Globally, it is estimated that approximately 43% or 250 million children younger than 5 years are at risk of not reaching their developmental potential [8]. Furthermore, there is evidence to suggest that an *inverse care law* is in operation, with children from disadvantaged backgrounds being least likely to engage with early detection initiatives, despite being at the highest risk of developmental delay [9-13].

Population health is largely defined by social determinants. Facilitators of disadvantage include living in a medically underserved area, being of an ethnic minority background, and having a lower socioeconomic status and fewer educational opportunities [14]. Policy bodies recommend universal developmental surveillance for all children younger than 5 years and targeted developmental surveillance for children in disadvantaged populations as best practice in early detection and breaking health inequities, respectively [8].

Developmental surveillance is defined as a flexible, longitudinal, and cumulative process whereby health professionals identify children who may be at risk of developmental delays via clinical interviews and observations. It also often involves the use of a standardized screening instrument at routine times in the first several years of a child’s life [15]. The use of standardized instruments to identify and refine a developmental risk or potential issue that emerges from surveillance is termed developmental screening [16]. Screening provides greater sensitivity and specificity than surveillance. It does not yield a diagnosis per se but rather is a process by which a child’s development may be identified as atypical compared with similar-aged children. This is different to developmental trackers or records. Child development apps or smart diaries, such as babyTRACKS (babyTRACKS team) and BabySteps (Beyond Blue & Queensland University of Technology Institute of Health and Biomedical Innovation), assist with caregiver monitoring but do not guarantee action on the part of the caregiver or the pivotal connection with a health professional should any developmental issue be identified [17,18]. Moreover, they typically only involve the motivated few who download the app. For example, Australian studies reported inconsistent use or follow-up of *The Blue Book*, a government resource distributed to all parents at their child’s birth to assist with monitoring their child’s development [19,20].

Developmental surveillance and screening are costly endeavors limited by the existing service infrastructure and resources. The increasing popularity and ownership of mobile technologies offer potential economies of scale, as it may be easier to perform surveillance for a larger number of people [21]. This improved reach, accompanied by possible clinical efficiencies in reduced human error and time, and improved cross-disciplinary communication and referral uptake via automated systems may be especially beneficial among disadvantaged communities where health knowledge and access to and engagement with services may be lowest [22-25].

Distinct from cellular telephony, mobile technologies with internet access include smartphones and ultraportable computers or tablet PCs, such as iPhones or iPads. Evidence regarding the implementation of web-based health interventions in maternal and child health is mixed [26]. Successful implementation depends on the usability of the intervention to both the deliverer (ie, the health professional) and the recipient (ie, the patient). If an intervention, or in this context a screening tool, is considered usable by the patient, then they are more likely to adhere to treatment recommendation and benefit from improved clinical outcomes. If a screening tool is considered usable by the health practitioner, it is more likely to be implemented as intended [27]. Usability can be thought of as encompassing 2 components [28]. The subjective component refers to users’ satisfaction with or acceptability of the tool, such as the perceived usefulness and ease of use. This is almost a necessary but not sufficient condition for the objective component, which pertains to the effectiveness of a tool.

### Objectives

This review aimed to (1) systematically search for and identify existing web-based programs that implement a developmental surveillance or screening tool and (2) appraise them for their usability (ie, acceptability and effectiveness) in the early detection of developmental delay in infants and preschool children, with particular consideration given to sample demographics implicated in health disadvantage.

## Methods

The review was registered with PROSPERO (CRD42019127894). The search strategy and inclusion and exclusion criteria were based on the following Participants; Intervention; Comparators; Outcome definition.

### Participants

The review considered studies that included children aged 0 to 6 years (ie, infants aged 0-2 years and preschool children aged 2-6 years).

### Intervention

The review included developmental surveillance or screening tools and methods that had the aim of assisting a health professional in identifying children at risk of developmental delay. The review focused on such practices delivered via the internet. It excluded cellular or telehealth, electronic health records, data management systems, or general computer or video use—unless they were used as part of a web-based system. Similarly, interactive computer systems designed to assist health professionals in making a diagnostic decision were only included if they had reached the application (ie, not design/algorithm) phase and were being implemented over a web-based system. Smart or robotic assessment toys were also excluded unless they were part of a web-based screening assessment.

### Comparators

Studies need not have had a control group for inclusion in the review. Comparators, if present, included paper versions of the web-based tool.

### Outcome

The review considered outcomes that reported the following:

- Information on the existence of a web-based developmental surveillance/screening tool or program, inclusive of study protocols, abstract proceedings, and dissertations.
- Original data pertaining to the user satisfaction with or acceptability of a web-based developmental surveillance/screening program, as a standalone tool or in comparison to a paper version of the cited tool.
- Original data pertaining to the effectiveness or ability of a given intervention to detect the risk of early (neuro)developmental delay. This included predictive specificity/sensitivity analyses, relative to the target diagnosis or as compared with a paper-based version of the tool. This included the risk of ASDs, communication disorders, developmental disabilities, and motor skills disorders. It excluded tools focused exclusively on detecting mental health disorders, such as attention deficit hyperactivity disorder, conduct or oppositional defiance disorders, or Tourette syndrome, or parent-child relationship issues, such as separation anxiety or reactive attachment disorder. The review excluded outcome variables related to medical disorders or syndromes such as low birthweight or preterm cardiac issues and single gene disorders such as cystic fibrosis and Down syndrome.

### Information Sources

Studies were identified through the following methods:

- Electronic databases were systematically searched: MEDLINE (Medical Literature Analysis and Retrieval System Online; Ovid platform), EMBASE (Excerpta Medica database; Ovid platform), EmCare (Ovid platform), CINAHL (Cumulative Index of Nursing and Allied Health Literature; EBSCO platform), PsycINFO (Ovid platform), and Cochrane Library (Wiley platform).
- Reference lists of included studies were checked.
- Expert consultation: experts in the developmental surveillance field were consulted to identify other articles for possible inclusion in the review.

### Search Strategy

The MEDLINE search strategy was developed using an iterative process of preliminary searches testing MeSH (Medical Subject Headings) and keyword search terms under the guidance of an academic librarian. New search terms were incorporated, as relevant papers were identified. The final MEDLINE search strategy is provided in Textbox 1. Once this strategy was finalized, it was adjusted to the subject headings, syntax, and operating systems of the other databases. The search was conducted in February 2019. No date or country limits were imposed upon the search (the date was set *organically* by the search for web-based programs), and only studies published in the English were included. Nonoriginal data, such as reviews, letters, opinions, or narratives, were excluded. A gray literature search for unpublished studies also included a Google search and a Google Scholar search with the key terms “(mobile OR electronic OR smart) in various combinations with (“developmental surveillance” OR developmental screening).” The first 100 sources of each search were reviewed.

**MEDLINE search strategy.**

(exp Neurodevelopmental disorders/ or exp language development disorders/ or Developmental disabilit*.tw. or Neurodevelopmental Disorder*.tw. or developmental status.tw. or developmental milestone*.tw. or developmental delay*.tw. or autism.tw. or autistic.tw. or delayed development.tw. or development disorder*.tw. or language delay*.tw. or language disorder*.tw. or speech disorder*.tw. or communication disorder*.tw. or Aspergers.tw. or language development.tw. or Developmental coordination disorder.tw. or Developmental dyspraxia.tw. or Delayed speech.tw. or child development.tw. or language impairment.tw. or abnormal development.tw. or developmental disorder*.tw. or speech delay.tw. or language disabilit*.tw. or delayed speech.tw. or learning disabiliti*.tw. or intellectual disabilit*.tw. or learning problem*.tw. or learning difficult*.tw. or learning deficit.tw. or learning impairment*.tw. or psychomotor-delay.tw or psychomotor-disorder.tw or psychomotor-impairment.tw or delayed-psychomotor.tw or motor-skills-disorder*.tw or motor-skills-delay.tw)

AND

(exp mobile applications/ or exp Decision Making, Computer-Assisted/ or mhealth.tw. or mobile.tw. or ehealth.tw. or smart.tw. or web-based.tw. or multimedia.tw. or computer-assisted.tw)

AND

(exp public health surveillance/ or exp Mass screening/ or exp early diagnosis/ or screening.tw. or surveillance.tw. or diagnosis.tw. or early identification.tw or early detection.tw)

AND

(exp infant/ or exp child, preschool/ or exp child/ or neonatal.tw .or child.tw. or infant.tw. or toddler.tw or preschool*.tw. or paediatric*.tw or pediatric*.tw. or nursery.tw. or kindergarten.tw.)

### Study Selection

**Figure 1:**
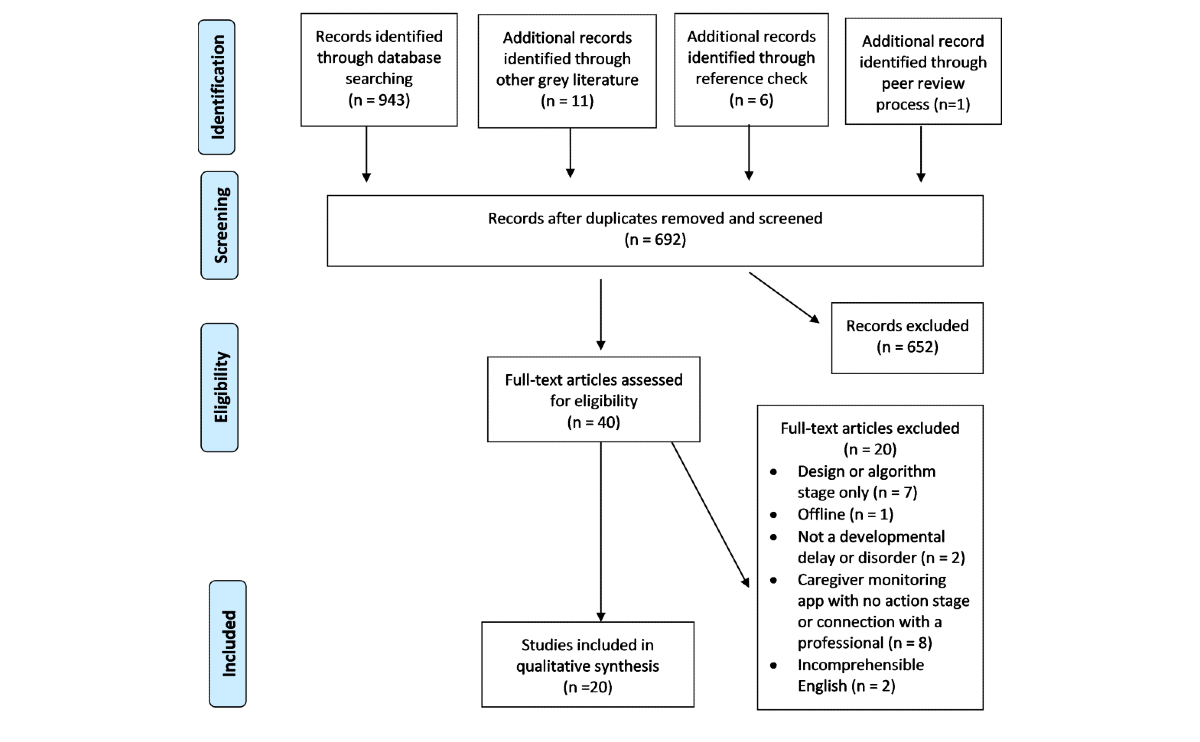
Preferred Reporting Items for Systematic Reviews and Meta-Analyses flow diagram.

Literature search citations were collated in a reference management software Endnote X8, and duplicate citations were removed. Authors JB and JK independently screened the titles and abstracts to determine whether a study met the general inclusion criteria. Each article was rated as include, exclude, or unclear. The full text of all articles classified as include or unclear was retrieved for formal review. Next, authors JB and JK independently assessed the full text of each study according to the predetermined inclusion criteria. Disagreements were resolved by discussion between the 2 reviewers and third author adjudication. The reasons for excluding studies were recorded. Review authors were not blind to the journal titles or authorship information of the studies. The search results are presented in a Preferred Reporting Items for Systematic Reviews and Meta-Analyses flow diagram (Figure 1).

### Assessment of Methodological Quality

Author JB assessed the methodological quality of included data-generating studies (ie, not abstracts or conference proceedings describing a device) using the Effective Public Health Practice Project (EPHPP) tool, which is recommended for quality assessment in systematic reviews inclusive of uncontrolled studies [29]. Paper quality was rated as *strong*, *moderate*, or *weak*, based on an overall assessment of study selection bias, design, blinding, control of confounders, data collection tools, and withdrawals or dropouts [30,31]. Author SO double coded the paper quality, with rater differences reconciled through discussion.

### Data Extraction

Study data were extracted using standard forms based on the Cochrane Effective Practice and Organization of Care Review Group. Data items extracted included study design, setting, study population (including number, age, and gender of participants), the device, disorder or type of delay being detected, and study outcomes relevant to the review question. Extraction also included social determinants identified by the PROGRESS-Plus acronym that impact disadvantage [14]. These were Place of residence, Race/ethnicity/language, Occupation, Gender, Religion, Education, Socioeconomic status, and Social capital. The *Plus* refers to contextual factors such as (1) personal characteristics associated with discrimination (eg, age and disability), (2) features of relationships (eg, smoking parents), and (3) time-dependent relationships (eg, leaving the hospital or other instances where a person may be temporarily at a disadvantage) [14]. Data extraction forms were first piloted and amended as necessary. Author JB performed the initial extraction on the included studies, which was then reviewed and refined by the author SO.

### Data Synthesis

A small selection of studies was anticipated; thus, a narrative approach was used to synthesize the results in relation to what web-based developmental screening programs exist and how acceptable and effective they are. The synthesis also considered social determinants related to disadvantage [32].

## Results

### Identified Studies

The search identified 20 eligible studies (Figure 1). Most web-based developmental surveillance programs were trials of online versions of established questionnaires, which based on automated risk scoring linked the child to a health practitioner for assessment either in person or by remote video. The gray literature and reference list searches identified several caregiver monitoring apps [17,18]. Most did not meet surveillance/screening criteria of embodying action to connect the user with a health professional should the developmental risk be apparent. For this reason, they were excluded at the full-text stage. The gray literature and reference list search also identified the prototype designs of smart toy screeners for ASD. However, except for 1 study, these were at the design stage or not yet web based [33]. All studies, except 1, were of adequate quality to be included in the review. A summary of the identified web-based measures is provided in Table 1. A total of 4 studies were at the design or protocol stage, and acceptability or effectiveness data were available in the remaining 15 studies. The data extraction table is available in Multimedia Appendix 1.

**Table 1:** A summary of identified web-based developmental surveillance/screening tools, including the targeted developmental delay and the availability of acceptability or effectiveness data.

Web-based screener	Delay/disorder	Number of papers	Design/protocol stage only	Acceptability data	Effectiveness data
M-CHAT-R/F^a^ (including Austim Barta)	ASD^b^	9^c^	No	Yes	Yes
Parent Evaluation of Developmental Status tools	Broad	3^c^	No	Yes	Yes
Child Health and Development Interactive System (including M-CHAT and Ages and Stages Questionnaire)	ASD/broad	1	No	Yes	No
Taipei II Pre-schooler Developmental Checklist	Broad	2	No	Yes	Yes
Cognoa	ASD	1	No	Yes	Yes
Mobile Autism Risk Assessment	ASD	1	No	No	Yes
Naturalistic Observation Diagnostic Assessment	ASD	1	No	Yes	No
Gades System (Denver Developmental Screening Test)	Language	1	Yes	No	No
Pirate Adventure Autism Assessment app	ASD	1	Yes	No	No
Smart Autism/Autism Express (including M-CHAT-R/F and Childhood Autism Spectrum Test)	ASD	1	Yes	No	No
Smart Toy Tower	Psychomotor	1	Yes	No	No

^a^M-CHAT-R/F: Modified Checklist for Autism in Toddlers, Revised with Follow-Up.

^b^ASD: Autism spectrum disorders.

^c^Glascoe [34] reported on both measures.

### Data Quality of Identified Studies

Accommodating for design limitations of the 20 studies, the overall modal quality was *moderate*, as based on the EPHPP tool. The study samples typically appeared representative of their target population, in that most studies were conducted during routine visits across public health clinics. However, some selection bias was introduced in studies conducted in academic clinics. Overall, there was an absence of data across studies pertaining to the percentage of selected individuals who agreed to participate. With the exception of 2 studies that implemented a controlled cross-over design [35,36], most studies were of a one-time assessment or cohort nature, which limited overall study design quality. Furthermore, caregivers or health professionals performing the assessment were necessarily unblinded to the *intervention* status because of the salient web versus paper format, or information about blinding was not reported. Assessment of components related to attrition rates or control of confounders was largely rated as *not applicable* because of most studies being of a one-time assessment or cohort design.

### What Web-Based Developmental Surveillance Programs Exist?

#### Web-Based Modified Checklist for Autism in Toddlers, Revised With Follow-Up

The most consistently used web-based developmental surveillance measure was the digital Modified Checklist for Autism in Toddlers, Revised with Follow-Up (M-CHAT-R/F) [37]. The M-CHAT-R/F is a two-stage screening tool designed to identify children aged 16 to 30 months who should receive an assessment for possible early signs of ASD or developmental delay. Parents score an initial set of 20 yes/no questions. Depending on the score, their child is deemed as *low*, *medium*, or *high* risk. Medium-risk children complete the second follow-up questionnaire, and the responses to this determine whether the child is referred for diagnostic evaluation or no immediate action is required. High-risk children bypass the follow-up questionnaire and are referred immediately for diagnostic evaluation. The web-based M-CHAT-R/F automatically directs parents to the appropriate follow-up questions, and health practitioners can access the results immediately.

The review also identified a paper on the web-based Child Health and Development Interactive System (CHADIS), a *package* of several screening instruments assessing a range of developmental, behavioral, and socioemotional issues [38]. The CHADIS administered the M-CHAT to children aged 0 to 3 years. A web-based M-CHAT-R/F was also used in a stepped developmental screening approach—in that it was implemented after the completion of a web-based broadband developmental screen [34].

A total of 4 web-based M-CHAT-R/F studies were conducted in America, with non-English–speaking participants typically excluded [39-42]. These studies did, however, report diverse cultural samples, including a significant proportion of Medicaid-insured families (a government program for persons whose income and resources are insufficient to pay for health care), across different socioeconomic statuses and urban and rural practices. Two identified conference proceedings reported trialing a smart M-CHAT-R/F app in Bangladesh with pictorial representations [43,44], whereas another trialed a web-based M-CHAT-R/F in a *low-risk* population in Sicily [45].

#### The Parents’ Evaluation of Developmental Status and Parents’ Evaluation of Developmental Status: Developmental Milestones

Three identified studies used the broad-screen developmental Parents’ Evaluation of Developmental Status (PEDS) tools for children aged 0 to 8 years [34,46,47]. The PEDS elicits parents’ concerns about their child with 10 open questions, such as Do you have any concerns about how your child understands what you say? and Do you have any concerns about how your child behaves? The PEDS: Developmental Milestones is a 6- to 8-item screener that assesses children’s skills across expressive language, receptive language, fine motor, gross motor, social emotional, self-help, and academic domains. The tools are available through a web-based portal, *PEDStestonlin*e. Automated scoring generates referral letters and take-home parent summary reports and identifies appropriate billing codes. A portal is also available through which parents can complete measures before their child’s health visit, and findings are sent to each clinic or provider without parents first seeing the results. The *PEDStestonline* also includes an option to deliver the MCHAT-R electronically.

The review identified 1 large-scale American implementation study of the *PEDStest online*, in which participating families were described as having *elevated psychosocial risk factors* [34]. The study reported that compared with census data, the sample was disproportionately poor, of an ethnic minority, non-English speaking, and had lower than average high school graduation rates. The review also identified the PEDS tools as a smartphone app for trial in South Africa. The trial was set in government Baby Wellness Clinics, and non-English–speaking caregivers were excluded [46]. Another South African trial of the application targeted vulnerable families who had children infected or affected by HIV/AIDS. The net monthly income of the majority of these families was less than US $155, and most households included more than 3 children [47].

#### Other Autism Spectrum Disorder Developmental Screening Tools

Most other identified web-based developmental screening tools were for ASD. One study described the development and evaluation of a *Naturalistic Observation Diagnostic Assessment (NODA)*. NODA is a web-based smartphone app that allows parents to collect in-home videos of their 2- to 6-year-old children’s behavior to support practitioners in completing a diagnostic assessment of ASD [48]. The paper described an associated web portal that permits practitioners to direct the in-home video collection, access the child’s developmental history, and conduct a remote diagnostic assessment by linking behaviors tagged in the videos to diagnostic criteria. Another study compared the effectiveness of a web-based screening tool called *Cognoa* for children aged 18 to 72 months, which integrated a 15-item parent report with the clinical ratings of brief video segments uploaded via parent’s smartphones to calculate ASD risk based on an automated score [49].

Bardhan described the framework of a cloud-based mobile app for virtual ASD screening, named *Smart Autism* in one paper and *Autism E*xpress in another [44,50]. It incorporated the questions of 3 different ASD screening tools for children aged 0 to 17 years. The tools relevant to the review target age were the M-CHAT-R/F and the Childhood Autism Spectrum Test, a 39-item, yes or no evaluation aimed at parents of children aged 3 to 11 years [51]. The caregiver enters their child’s birthdate, and the app selects the appropriate questionnaire. If ASD is suspected based on the automated scoring of the caregiver’s responses, a video stream is sent to the user’s device to play in front of the child. The camera within the mobile device records the child’s reactions and uploads the video to the cloud for an expert to observe. If the expert suspects that the child might have ASD, the nearest autism resource center (ARC) is notified, and the ARC address is sent to the user. The expert observes the child in person at the ARC and provides their diagnostic decision in the cloud.

Two other studies described web-based ASD screeners for an age range inclusive of, but broader than, the study focus. One tested the sensitivity and specificity of a new Mobile Autism Risk Assessment (MARA) for children aged 16 months to 17 years. The MARA was a 7-item parent report of a child’s communication, social skills, and behaviors, to triage those at the highest risk of ASD. It is completed electronically on an iPad, computer, or any other device connected to the internet and is automatically scored [52]. In the trial of MARA, non-English–speaking caregivers were excluded. The design of a Pirate Adventure Autism Assessment app was also identified in the review. The app adapted well-established affect recognition and Theory of Mind tests (ie, Smarties Tube and Sally Ann tests) to diagnose ASD via an engaging pirate adventure story line for children aged 6 to 7 years [53].

#### A Developmental Language Delay Screening Tool

One identified paper detailed the development of a web-based clinical decision support solution, termed the Gades System, to support the efficient detection of language disorders among children aged 0 to 6 years in routine visits to pediatricians in primary care. It evaluated sensory reception, speech perception, speech production, and pragmatic language and had foundations in the established *Denver Developmental Screening Test* [54]. Negative responses to questions termed *Alert Milestones* recommend a follow-up visit to the pediatrician within 3 months to reevaluate the level of language acquisition, whereas negative responses to questions categorized as *Alarm Milestones* suggest a direct referral to a specialist in language disorders.

#### A Psychomotor Development Delay Screening Tool

The review also identified a *smart toy* screener for detecting children’s psychomotor delays through natural interaction with toys. Specifically, the paper described the design, development, and validation of a tower with 5 stackable cubes embedded with a sensor data collector module [55]. As toddlers aged 23 to 37 months made the tower, sensors in the cubes sent data to a collector module through a wireless connection for the automatic detection of psychomotor developmental delays.

#### General Developmental Screening Tools

Two identified studies described the development and validation of a web-based version of the Taiwanese government–established *Taipei Pre-schooler Developmental Checklist second version* (Taipei II) for early detection of developmental delay [35,36]. It provides 11- to 13-item checklists for 13 age groups from 4 months to 6 years, related to easily observable behaviors or skills in the domains of motor, cognition, language/communication, and emotion/social development. The Taipei II specifies the use of pictures instead of text to avert literacy barriers.

The web-based CHADIS screening assessment for developmental, behavioral, and socioemotional issues also included the *Ages and Stages Questionnaire* (*ASQ)* for children aged 0 to 3 years [38]. The parent-reported ASQ assesses a child’s personal, social, gross motor, fine motor, problem-solving, and communication skills [56].

### How Acceptable Are Existing Web-Based Developmental Surveillance Programs?

#### Implementation Preferences

Most trials of the web-based screeners for ASD or broad developmental delay were conducted out of pediatric or general practitioner community clinics, public health centers, or university specialty clinics and incorporated into routine baby wellness or child checks [34-42,45,46,49,52,53]. One of the South African PEDS tool studies was incorporated into a home-based health visitor service [47]. The Smart Toy Tower or Gades System screeners for psychomotor or language delay were based in nursery schools [54,55].

For studies beyond the protocol stage, namely, the digital M-CHAT-R/F and PEDStestonline papers, implementation typically took the format of parents completing the screener on a provided netbook, computer, or iPad in the clinic waiting room before meeting a health practitioner for a routine child checkup. The results were immediately provided to the practitioner to guide their action during the family’s appointment [39-42]. An additional implementation method for the PEDStestonline was to give the parents an appointment reminder to complete the online screening assessment before the next scheduled visit using a link provided [34].

In an evaluation of the implementation of the PEDS tools across 79 clinics that frequently used the screeners (online and offline), 24 clinics used the web-based parent portal version and collectively screened 2086 of the total 20,941 (9.96%) eligible children [34]. Many clinics were private practices, and parents accessing the portal were significantly more likely to be English speaking. The clinics with the highest web-based portal uptake provided computers in the waiting room and had attendants assist parents with using the computers, read the questions aloud for families with limited literacy, or entertain the children so that parents could complete the screens undisturbed [34]. The clinic with the lower rates of portal use utilized the appointment reminder approach. The review also identified a capacity to train community health workers in South Africa in the use of a smartphone app of the PEDS tools [46,47].

The CHADIS assessment incorporating the M-CHAT and ASQ was completed by parents 2 weeks in advance of a child welfare visit. The assessment results were communicated to the practitioner and used to determine what type of visit the family pursued. Healthy development scores determined an *electronic visit (e-visit)* by email exchange, with or without a brief face-to-face encounter with a practitioner for a physical health checkup [38]. Scores that indicated a concern necessitated an *extended encounter* with a health professional.

#### Uptake Efficiency

In trials of the M-CHAT-R/F, web-based screening was reported to be an efficient and acceptable method over paper-based screening. For example, there was a 59% increase in the number of toddlers screened per month when web-based screening was introduced at an urban pediatric clinic [39]. The web-based method also resulted in only 3% of missing data at the follow-up stage, compared with 35% of missing data utilizing the paper-based method [39]. Similarly, the implementation of a smart M-CHAT-R/F at a primary care clinic occasioned a 38% increase in the accurate documentation of screening results (from 54% to 92%), and a 60% increase in appropriate action for children screening positive for a delay (from 25% to 85%) [42]. When continued use of the paper form did occur, it was reported to be primarily when multiple patients arrived and there were insufficient computers to screen simultaneously [41].

#### Practitioner Preferences

More than 90% of the participating physicians indicated that they preferred the web-based method of the M-CHAT-R/F over paper forms [41]. They agreed that it improved their clinical assessment of ASD risk and that the automatically generated score made the M-CHAT-R/F easier and faster to use. Furthermore, primary care practitioners were shown to successfully administer the web-based M-CHAT/RF after a 10-min interactive multimedia demonstration [42]. For the CHADIS, of which the M-CHAT and ASQ were a part, the sample of 7 providers believed that the online format helped them to focus on the family visits and that they would continue to use the web-based system [38]. For Cognoa, the app that integrated parent report and video ratings for ASD screening, clinicians reported the video component to be the most helpful. In addition, 88% of physicians found the automated summary report helpful, and 76% of physicians found that the report and videos alerted them to look for particular behaviors [49]. Clinicians also rated the NODA as clinically useful in performing an ASD diagnosis [48].

#### Caregiver Satisfaction

Higher rates of parental satisfaction were also reported with the iPad versus paper administration of the M-CHAT [40]. Most parents did not require help to complete the computerized M-CHAT, and most parents did not experience anxiety when they viewed the result of their M-CHAT on the iPad screen [40]. Caregiver satisfaction with, and preference over its paper version, was also reported for the Taipei II [35,36]. Similarly, parents were reportedly easily able to use the NODA app without prior training [48]. Regarding the CHADIS, which was inclusive of the M-CHAT and ASQ, three-fourths of parents reported that the online previsit assessment improved their child welfare visit. However, nearly one-fourth of parents found the web-based assessment difficult to use [38].

### How Effective Are Web-Based Screeners in Detecting Developmental Delay?

One identified study found that the web-based format of the earlier M-CHAT lowered both (1) false-positive screens (through the use of the M-CHAT follow-up if the child was deemed at risk after the first-level screen) and (2) false-negative screens (through the elimination of human scoring errors) [40]. However, in a later study comparing a paper-based version with the web-based version of the updated M-CHAT-R/F, no significant differences were observed in screen-positive rates or total scores [39]. Similarly, high positive and negative correspondence were demonstrated between the paper-based tools and smartphone PEDS tools in the South African trial [46].

Excellent agreement was also observed between the text and multimedia versions of the Taipei II [35], and the MARA reported 90% and 80% sensitivity and specificity in detecting ASD, respectively [52]. The Cognoa was compared with other often-used paper ASD screeners, including the M-CHAT-R/F. It was shown to accurately identify ASD in 71% of children aged 18 to 72 months. The overall specificity in detecting ASD (0.62) was significantly higher than the other measures [49]. The Gades System based on the Denver Developmental Screening Test demonstrated acceptable reliability for identifying language delay in children aged 0 to 3 years (97% agreement), and 67% agreement for children aged 4 to 6 years [54].

## Discussion

### Principal Findings

This was the first paper to identify existing web-based developmental surveillance or screening tools and summarize their acceptability and effectiveness in the early detection of developmental delay in infants and preschool children. It is timely, given the increased focus on electronic health and applications in health care [22-25]. The search identified 20 eligible studies. The majority (n=9) of studies used web-based versions of the established M-CHAT-R/F screener for ASD or PEDS tools for detecting broad developmental delay. Only 2 studies screened for language or psychomotor delay [54,55]. This indicates a lag in the web-based movement regarding these developmental domains. Implementation typically took the format of parents completing the screener on an iPad in the waiting room before a routine baby wellness checkup at a community health clinic, with the automatically scored results immediately provided to the treating practitioner to guide their action during the visit.

Web-based versions of developmental screeners demonstrated improved acceptability in primary care relative to their paper version. Caregivers preferred web-based screeners primarily for their ease of use and time efficiency [40,41]. However, families were less positive about the ease of use of the CHADIS. This could be because it involved an assortment of questionnaires, rather than 1 individual questionnaire [38].

Clinicians also found web-based versions of screeners easier and quicker to use. The clinical implication of this is readily apparent when one considers that a key factor for not using the follow-up screen of the M-CHAT/RF is administration time [41]. Specifically, studies reported improved screening rates, accurate documentation, and appropriate follow-up action for children screened positive when the web-based versions were used [39,41,42]. The M-CHAT follow-up screen is most helpful in reducing high initial screen false-positive rates [57]. In this way, it could have an indirect positive impact on the effectiveness of the screener. One identified study found that the web-based M-CHAT lowered both false-positive and false-negative screen rates [40]. However, another study found no significant difference in screen rates for the paper-based version versus the web-based version of the M-CHAT-R/F [39].

Certainly, evidence for the effectiveness of the web-based developmental screeners was minimal. Satisfactory sensitivity and specificity rates were reported for the MARA [52], and a marginally satisfactory specificity rate was reported for the Cognoa [49]. The paper-based M-CHAT-R/F and PEDS tools endorse strong psychometric properties and a wealth of effectiveness data [37,58]. However, it cannot be assumed that this translates over to web-based versions. Future research would benefit from examining whether the improved acceptability of web-based screeners reported in this review can translate into quicker identification of a potential developmental delay, earlier referrals, and ultimately earlier age of diagnosis.

### Implications for Disadvantaged Communities

One of the biggest barriers to screening access is not speaking English. The screening tools developed in Bangladesh and Taiwan used pictures to avert literacy barriers [35,36,43,44]. The M-CHAT-R/F and PEDS tools are also well placed to address this barrier in that they are available in numerous different languages. However, where non-English speakers were included in a study, non-English–speaking caregivers were still less likely to complete the web-based version versus paper-based version of the screener. Furthermore, it was in clinics that employed attendants to read questions aloud for families with limited literacy that reported the highest web-based portal uptake [34].

The reported improvement in follow-up rates for web-based versus paper-based screeners may help reduce sociodemographic barriers relevant to developmental screening, in that higher rates of incomplete follow-ups are associated with lower maternal education [59]. The finding that community health workers could be trained in a smartphone app of the PEDS [46,47] also holds promise in increasing engagement rates in disadvantaged communities by removing some of the logistical barriers associated with screening access. The CHADIS was innovative in trying to capitalize on web-based screeners to minimize the resource demands involved in developmental screening via triaging the intensiveness of the routine child welfare checkup [38]. Although there is intuitive validity in assigning children with scores indicative of healthy or delayed development to an email-based or face-to-face welfare visit, respectively, most parents reported not wanting to see the e-visit replaced by a regular visit [38]. Certainly, experts reported that it was the video (ie, face-to-face) component of the Cognoa app that was most helpful in screening for ASD as compared with the parent report [49].

Indeed, the review identified several emerging smart applications that integrated caregiver report with videos for remote viewing by experts, such as the Cognoa and SmartAutism or AutismExpress [44,49,50]. Although such designs put an onus on the parent to initiate the developmental checkup, which may be less likely in disadvantaged communities [19,60], they do present as promising opportunities for accessing rural and under-resourced communities.

Several studies purposefully targeted culturally diverse or socially disadvantaged communities or families [34,46,47]. This is in keeping with policy body recommendations for targeted developmental surveillance. However, it challenges generalizability. Future research explicitly comparing the implementation of web-based screeners across disadvantaged and nondisadvantaged groups would facilitate a better understanding of the needs of disadvantaged communities relevant to screening inequities. Moreover, most identified studies were based in high-income countries; yet, it is in low- and middle-income countries that estimates of children younger than 5 years not reaching their developmental potential are the highest [8].

### Limitations

The review was limited by its stringent exclusion criteria, meaning that some promising web-based tools in production were beyond the scope of the study. Furthermore, the inclusion of some studies that targeted a wider age range than the study focus may have added noise to the data [52,53], and raises the debate of where developmental screening ends and broader general assessment begins.

### Conclusions

In summary, although the research is limited as to whether a web-based system is necessary for developmental screening, this review clearly highlights the important time and follow-up efficiencies that can facilitate policy body recommendations for universal developmental surveillance. Societal reliance on smart technology is increasing. It is hoped that increasing traction in web-based developmental screeners will continue as a possible means to promoting the valuable earlier detection of developmental delay in the infant and preschool years.

## Appendix

Multimedia Appendix 1 Data extraction table.

## Abbreviations

ARC: autism resource center
ASD: autism spectrum disorder
ASQ: Ages and Stages Questionnaire
CHADIS: Child Health and Development Interactive System
EPHPP: Effective Public Health Practice Project
e-visit: electronic visit
MARA: Mobile Autism Risk Assessment
M-CHAT-R/F: Modified Checklist for Autism in Toddlers, Revised with Follow-Up
NODA: Naturalistic Observation Diagnostic Assessment
PEDS: Parents’ Evaluation of Developmental Status
