# Supplementary material for: The Acceptability and Effectiveness of Web-Based Developmental Surveillance Programs: Rapid Review
Source: JMIR Mhealth Uhealth. 2020 Apr 23;8(4):e16085. doi: 10.2196/16085 (PMC7206511; doi:10.2196/16085)
Supplement: Multimedia Appendix 1 [file mhealth_v8i4e16085_app1.docx]

| **#** | **Title** | **First authors** | **The Device/** | **Delay/Disorder** | **Age** | **Aim/objectives** | **Design** | **Setting** | **N** | **Gender** | **Relevant outcomes** | **PROGRESS-Plus** |
| --- | --- | --- | --- | --- | --- | --- | --- | --- | --- | --- | --- | --- |
| 1. | Smart Autism - A mobile, interactive and integrated framework for screening and confirmation of autism | Al Mamun, Bardhan et. al. 2016 | Cloud-based; Automated. 3 steps:   1. Evaluates the responses of pictorial based screening questionnaire through mobile application. 2. If ASD is suspected, the child watches a video; its reaction is recorded and uploaded to the cloud for remote expert assessment. 3. If ASD still suspected, child referred to the nearest Autism Resource Center (ARC) for in-person assessment. | ASD | 0-17 years | **DESIGN STAGE ONLY.** |  |  |  |  | No outcome data yet | Bangladesh |
| 2. | Autism Barta - A smart device based automated autism screening tool for Bangladesh | Bardhan, et al. 2016 | - Adapted **M-CHAT** - Informs user, stores responses in an online database and suggests nearby ARCs for confirmation and intervention. | ASD |  | **CONFERENCE PROCEEDING - DESIGN STAGE ONLY.** |  |  |  |  | No outcome data yet | Bengali version of the M-CHAT integrated with pictorial representation. |
| 3. | Autism Express - A cloud-based framework for autism screening, confirmation and intervention | Bardhan et al. 2016 | - Cloud-based automated system using a 3G smartphone with internet availability - Adapted **M-CHAT-R** for (16-30/36-month-old toddlers), and Childhood Autism Spectrum Test **(CAST)** for 3-11 year olds (plus other questionnaires for older age groups).   Screening:   1. Inbuilt algorithm selects appropriate questionnaires and generates automated decision on whether child susceptible to ASD, based on parental answers. 2. In the mobile device child is shown videos of some activities. The camera of the mobile device records the expression and activities of the child and sends it to the cloud.   Virtual Assessment:   1. Expert remotely observes child’s video and determines whether child has ASD symptoms or not. Expert then sends his decision in the cloud. 2. If expert suspects that the child has ASD, then address of the nearby ARC is sent to the user and experts of that ARC informed about the child.   Actual Assessment:   1. In the ARC, the expert physically observes the child and gives decision in the cloud. 2. If the decision is YES, then the child has ASD. If the child does not have ASD, then the record is kept on hold for 3 months before starting from beginning of the *Screening* step again. User gets the confirmation message in the smart device. | ASD |  | **DESIGN STAGE ONLY** | . |  |  |  | No outcome data yet | Bangladesh |
|  | The Use of Internet-Based Technology to Tailor Well-  Child Care Encounters | Bergman, Beck, et al. 2019. | The **Child Health and Development Interactive**  **System** **(CHADIS)** incorporates an internet-based developmental, behavioural, and socioemotional screening assessment completed by the parent two weeks before a child welfare visit. The screeners relevant to the review include the:   - **M-CHAT** - **Ages & Stages Questionnaire (ASQ)** – a parent completed questionnaire that assesses personal social, gross motor, fine motor, problem-solving and communication skills.   The results are communicated to the practitioner and used to determine which of three types of visit the parent proceeds with:   1. E-visit - This is a fully   electronic visit in which the  screening tests document  normal development and parental concerns can be addressed via e-mail exchange.  2. E-visit with brief provider visit - This visit is for the  family that demonstrates normal results for the screening tests but needs to come to the office for a physical health check (e.g. immunizations, growth measurements). The brief visit assumes that the information-gathering  has been accomplished before the visit through CHADIS and e-mail.  3. Extended encounter – This visit is for the child who  has issues that require more attention and who  may need the involvement of other health care professionals. | ASD / Developmental delay | 0-3 years for relevant questionnaires | To evaluate the feasibility and acceptance of  a new internet-based developmental and behavioural screening model. | Cohort.  Non-randomised. | Two practices within a  health maintenance organization  serving 100 000 children. | 78 families;  7 providers | Not provided | - 10 families participated in the e-visit, 25 families participated in the e-visit with a brief provider visit; and 15 families participated in the extended visit. - 75% of parents thought that the online pre-visit assessment improved or very much improved the child welfare visit. - 12% of parents found the online assessment somewhat or very difficult to use. - All parents found the e-visit or the e-visit with brief provider visit acceptable or very acceptable, compared with a standard child welfare visit. - Most parents reported not wanting to see the e-visit replace a regular visit. - All providers thought that the new model helped focus the visit and that they would continue or definitely continue to use the model. | - Denver, Colorado, America - Metropolitan area |
| 4. | Implementation of Web-Based Autism  Screening in an Urban Clinic | Brooks, Haynes, et al. 2016 | Initial **M-CHAT-R/F** scored automatically.   - Scores 0-2 = low risk and screening complete. - Scores 3 to 7 = moderate risk, and follow-up questions administered automatically. Children who continued to demonstrate risk on 2 + follow-up items considered screen positive. - Children whose initial screening scores were 8 and higher considered screen positive, and follow-up questions bypassed.   Physicians had access to screening results immediately. Research personnel received the web-based screens through secure online portal and then contacted families who screened positive to offer diagnostic evaluations at University. Paediatricians indicated ASD concerns by adding a note in the electronic portal. | ASD | 18- and/or 24 months well child visits  (M=  22.43; SD= 3.65) | Comparison of paper vs web-based version of M-CHAT-R/F | Cohort.  Non-Randomised | Urban low-income paediatric clinic serving a culturally diverse population | 2557 | 47% Female and 52% male. 1% no response | - Web-based screening led to a 58.5% increase in the number of toddlers screened during the first 9 months of implementation: Web (M = 56.78, SD = 15.88) completed at a significantly higher rate than paper (M = 35.82, SD = 10.75), t(64) = −5.07, P < .001, η2 = .29. - No significant differences in: screen-positive rates or M-CHAT-R total scores - Significant difference in missing data at Follow-up stage χ2(1, n=427) = 32.11, p< .00.: Paper = 35.1% missing; web = 3.1% | - Atlanta, Georgia - 87% African American. - Average years of maternal education = high school diploma (M=12.37, SD = 1.32). - Non-English-speaking excluded |
| 5. | Use of a Digital M-CHAT-R/F to Improve Quality of Screening for Autism | Campbell et al. 2017 | **M-CHAT-R/**F custom-built tablet application that presented instructions and response items to parents. Password protected. Automatically scored answers and presented and scored follow up questions for secondary screening of medium risk results (score of 3–7).  Nurses used a password to view results and a secure network to wirelessly print a score report that provided the calculated risk category as well as text of the screen positive questions both from the initial screen and the follow up questions. The score report was provided to the physician before he or she entered the room in order to give the physician the opportunity to use screening results and screen positive questions to guide their action and the clinical interview. | ASD | 16-30 months old presenting at the well-baby clinics. | To assess changes in quality of care for children at risk for ASD due to process improvement and implementation of a digital screening form.   - 1. Can digital smart form technology be effectively used to implement the M-CHAT-R/F secondary follow up questions in routine care?   2. Does use of this technology increase fidelity of implementation, accurate documentation, and appropriate action?   3. Does use of this technology increase the acceptability of ASD screening to physicians in a primary care practice? | Cohort | Duke Children’s Primary Care clinic | 1,191; 99% of children screened. | 50% males | - Mean of 72% of children were screened with the smart form per month, with the remainder receiving paper forms. - 62% of children were screened with the digital form in the first month with paper forms being used due to technological challenges or when multiple patients required screening simultaneously. By the final month of the intervention, 80% of screening occurred using the digital form, with the paper form used when multiple patients required screening simultaneously. - Proportion of children screening positive with accurate electronic documentation, significantly increased from a mean of 54% to 92% (38% increase, 95% CI [14%, 64%]), and appropriate action increased significantly by 60% in the intervention period (95% CI [35%, 85%]). - In the intervention period, of the 22 children with appropriate action, 17 were presumed false positives ruled out with use of automated secondary screening and 5 children scored high risk or remained positive after automated secondary screening and were referred for evaluation. This amounted to an overall positive rate of 1.9% (5 previously identified + 5 newly identified out of 529 children studied) in the intervention period. - Prior to the intervention, only 25% used follow up questions and 56% reported routinely referring children for developmental evaluation after a positive screen but this increased to 100% after the intervention - 90% reported preference for the digital method and stated that this improved their assessment of ASD risk. - Time was reported as the main barrier for not using the secondary screen, and the automatically generated M-CHAT score report was perceived to be easier and faster. - Accurate documentation in the electronic medical record of screening results increased from 54% to 92% (38% increase, 95% CI [14%, 64%] and appropriate action for children screening positive increased from 25% to 85% (60% increase, 95% CI [35%,85%]). | - 42% Caucasian. - 28% African American - 7% Asian - 6% Hispanic/Latino - 4% Multiracial |
| 6. | The Design and Validation of a Child Developmental  e-Screening System | Cheng, Chang,  et al.  2017 | Multimedia e-screening system based on the text version **Taipei II** (Taipei Preschooler Developmental Checklist 2nd version) | Broad developmental delay | Primary caregivers of 6, 12, 18 and 30 month olds.  M = 29.8 ± 20.6 months (range 4–30months). | Validation and usability of a multimedia version of Taipei II, relative to paper version | Cross-over design with 2-week washout period. Random assignment. | Local public health centers in Taipei, Taiwan. | 120 | 38 male; 82 female  M age of 31.37 ± 7.52 years (r 23–70 years) | - 2-week Test-retest reliability was high (r = 0.93). - Excellent agreement between text-based and multimedia versions - 98% participants preferred the multimedia version or had no preference. 80% (n = 96) very comfortable and 20% (n = 24) were somewhat comfortable | Taiwan  Use of pictures over text to overcome literacy barrier |
| 7. | A Multimedia Child Developmental Screening Checklist: Design  and Validation | Cheng & Chen et al.  2016 | Web-based multimedia version of the current **Taipei II** developmental screening tool (see reference above) | Broad developmental delay | Primary caregivers of 4 months to 6 years  M = 29.8 (SD 20.6, r 4-84) months | 1. Design and develop a Web-based multimedia version of Taipei II  2. Investigate the measurement equivalence to the paper-based version. | Cross-over design with 2-week washout period.  Random assignment. | - Three paediatric and family physician clinics - Six local public health centres | 390 | 104 male, 286 female M age  33.35 (SD 6.71, r 23-70) yrs | - Excellent reliabilities for all age groups for cross-mode similarity (mode ICC range 0.85-0.96) and 2week test-retest reliability (r=.93). - Mean usability score for all the test items was 4.80 (SD 0.03), indicating that users were satisfied with their multimedia website experience; and 97.9% (382/390) preferred the Web-based version to the paper version | Northern Taiwan  Use of pictures over text to overcome literacy barrier |
| 8. | Clinical Evaluation of a Novel and Mobile Autism Risk Assessment. | Duda, Daniels et al. 2016 | The **Mobile Autism Risk Assessment (MARA)** Electronically administered, 7-question online screen to triage those at highest risk for ASD.  Queries a child’s communication, social skills, and behaviours. There are 4–5 answer sentence choices available for each question, as well as the option of ‘‘not applicable’’. The set of answers is run through a machine learning model that uses an alternating decision tree algorithm.  The model was trained on 891 ASD cases and 75 non-ASD controls from the Autism Genetic Resource Exchange. Finds autism behaviours measured by Autism Diagnostic Interview-Revised that predict known class values. | ASD | 16 months–17 years  median  age was 5.8 years | Evaluation of the MARA compared to: the Bayley Scales of Infant and Toddler Development, Third Edition, Differential Ability Scales; Second Edition of the Wechsler Intelligence Scales for Children, Fourth Edition; and the Vineland Adaptive Behaviour Scales, Survey Interview Form or Autism Diagnostic Observation Schedule if suspected. Finial clinic decision on DSM-5. | Cohort. | Developmental-behavioural paediatrics clinic of a large academic medical centre. | 222 | 76.1 % were male | - Sensitivity in detecting ASD was 89.9 % [95 % CI = 82.7–97] - Specificity was 79.7 % [95 % CI = 73.4–86.1] - (31%) received a clinical diagnosis of ASD. | - Massachusetts, USA - Written at a 7.9 grade reading level - Non-English speaking caregivers excluded - Most participants had an intelligence/ developmental quotient score >85 |
| 9. | Smart Toy to Enhance the Decision-Making Process at  Children’s Psychomotor Delay Screenings: A Pilot Study | Garcia, Ruiz et al. 2017 | **The EDUCERE Developmental Delay Screening System** on a mobile app for a table**t:**  A tower with five stackable cubes (embedded with sensor data collector module and a smart system for detection of developmental delays). As toddlers make the tower, sensors in the cubes send data to a collector module through a wireless connection. | Psychomotor development delay | 23-37 months (M=29.02; SD 3.81) | The design, implementation, and validation of an EDUCERE smart toy aimed to automatically detect psychomotor development. | Four experts in child psychomotor development  viewed the recordings. Each expert randomly viewed half of the videos and two experts individually rated each video. Thus, two experts scored each child’s performance independently. | Public nursery schools | 65 | 32 boys; 33 girls | - High inter-rater reliability (intraclass correlation 0.961, 95% CI 0.937-0.967), - A factor analysis showed three factors (trembling, speed, and accuracy), accounted for 76.79% of the total variance, but only accuracy (p=.001) and speed (p=.002) were predictors of performance. Trembling (p =.79), did not have a significant effect on this dependent variable. | Spain |
| 10. | Evidence-Based Early  Detection of  Developmental-Behavioural  Problems in Primary Care: What to Expect and How to Do It | Glascoe, 2015 | **PEDStestOnline -** includes Parents’ Evaluation of Developmental Status (PEDS), PEDS: Developmental Milestones (PEDS: DM), and M-CHAT.   - Automated scoring generates referral letters and take-home parent summary reports and identifies appropriate billing and procedure codes. - Parent-portal also available through which parents can complete measures before the visit but do not see results. Instead, findings are sent to each clinic or provider. - By design, PEDStestOnline users cannot use the M-CHAT (a narrow-band screen focused on identification of ASD) unless PEDS and/or PEDS: DM are used first. | Developmental delay/ASD | Birth to 8 years of age | - Inform clinicians about what to expect when using quality tools, including provision of information on identification rates by age, patient mix, and well-visit uptake. - Describe various implementation methods used by clinics. | A random sample of 22 (out of 79) practices were scrutinized. | 79 clinics total: Private practices in general paediatrics  and family medicine (N= 55 serving 14,698 families);  public health department and community  health centres (N = 14 serving 2,847 families) outpatient  teaching-hospital continuity clinics (N =3, serving 1,298 families)  and other services such as emergency  departs or nonemergent crisis call centres,  (N = 7 serving 2,103 families). | 20,941  Screened |  | - PEDS Online parent portal to complete screens prior to encounters was used by 24 (15 private practices and 5 community or public health clinics) of the 79 clinics covering 2,086 children (10% of the 20,941). - Of the 24 clinics, 20 used PEDS Online with 56% to 100% of families; the remaining 4 used it less than 35% of the time. - Clinics encouraged the portal use in two ways: (i) Having a waiting room computer kiosk or providing parents with tablet computers at check-in so complete screens in the waiting room on the day of the encounter; or (ii) Giving parents an appointment reminder card, including information on how to log in to the PEDS Online website together with a request to complete screens before the next scheduled visit. - Complete printed copies of screens in waiting rooms (74% of all families, N = 15,548/20,941). After parents completed measures, responses were entered by clinic staff into PEDS Online to obtain results. - Of the two approaches, clinics with the highest uptake on portal usage were those providing computers in the waiting room (56%, N = 1169/ 2086)*. The four clinics with lower rates of portal usage (44%, N = 917/2,086) used the appointment reminder approach.   * waiting-room attendants helped parents use computers, probed literacy, read questions aloud for families with limited literacy, entertained children so parents could complete screens undisturbed. | USA   - Purposefully selected clinics that provide to diverse SES and language backgrounds; - Parents accessing the portal were more likely to be English-speaking (OR = 3.2, 95% CI = 1.61–6.32, p < .0001) Otherwise no parental differences. - Participating families had elevated psychosocial risk factors and were disproportionately poor, ethnic minorities, non-English speaking, and had lower than average high school graduation rates (compared with U.S. Census Bureau data). - Of parents, 69% completed high school (compared with 84% nationally); 30% had incomes below poverty guidelines (versus 24% nationally); 34% were non-English speaking (compared with 12% nationally); 31% were Latino (compared with 17% nationally); and 50% were White (compared with 70% nationally). The incidence for other ethnicities was similar to Census Bureau data: 13% were African American and 6% were Asian, American Indian, or Pacific Islanders. - Clinics, even those serving families with limited education or lack of facility with English, found a variety of ways to make use of online screening services. |
| 11. | Screening Children for Autism  in an Urban Clinic Using an  Electronic M-CHAT | Harrington et al. 2013 | Electronic **M-CHAT** on the iPad. Parent completed screening questions. | ASD | 16 - 30 months old;  M = 22.1 months | 1. Examine the implementation of the electronic format of the M-CHAT, through the use of an iPad.  2. Decrease the false at-risk screen rates seen with the paper screens, streamline the screening process by eliminating the need for follow-up phone calls, reduce financial cost and staff time of administrating  the screen, and improve parental satisfaction with the screening process | A retrospective component of  patients screened using the paper M-CHAT for  a 6-month period prior to the implementation of the electronic M-CHAT,  acted as the control group for the  prospective component. | General Academic  Paediatrics outpatient practice located at The Children’s  Hospital of The King’s Daughters. | Of 213 patients approached, the parents/guardians of  176 (83%) patients agreed.  197 paper version. |  | - Most parents (170, 97.14%) did not require help to complete the computerized M-CHAT once they had started. - Most parents (166, 95%) did not experience anxiety when they saw the result of their M-CHAT on the iPad screen. - For those who had previously completed the paper M-CHAT (92), 71, 78% preferred the iPad version, but 22% still preferred the paper version. For overall experience, out of possible choices of “poor,” “fair,” “good,” or “excellent,” the most popular responses were (in order) excellent (137, 78%), good (37, 21%), fair (2, 1%). - Time take to complete the online version was quicker. - 17% of the paper M-CHATs that were incorrectly scored = one-third of them resulted in an incorrect final result - all of which were incorrectly scored as at risk. The electronic format lowered both false at-risk screens and false not-at-risk screens. Specifically, through using the electronic format of the M-CHAT, 5 children (3%) out of 176 were flagged as being at risk for ASD. This was significantly different to using the paper M-CHAT, where 20 children (10%) out of 197 were flagged as being at risk for ASD. | The outpatient practice has approximately 30 000 visits annually and the patient population includes low SES  predominantly urban, Medicaid enrolled, African  American patients. |
| 12. | Pirate adventure autism assessment app: A new tool to aid clinical assessment of children with possible autistic spectrum disorder | Jordan, Farr et al. 2018 | **Pirate app** involved storyboards built into the tool incorporating identified tests, adapted into the context of a pirate adventure story line. Tests of affect recognition included the ability to match facial expressions, identify appropriate situational facial expression, and short-term recall of facial expression. First Order Theory of Mind tests also included adapting the “unexpected contents (Smarties tube) task and Sally Ann tests, and Second Order Theory of Mind tests. The “Strange Stories” exploring the child’s ability to recognise and explain the use of sarcasm and a lie were added. | ASD | 6-year-old; 7-year-old girl | To determine if the pirate app was a useful adjunct to parental history and school questionnaire obtained at initial clinic, in determining the need for the child to proceed to a diagnostic assessment. |  | Three local Child Development Centres; two at initial appointment, in one at a diagnostic clinic. |  |  | - Diagnoses consistent with clinicians. - It provides information about the child’s patterns of thinking; equivalent to psychometric testing used in full diagnostic assessment, and when used together with parental history and school questionnaire can help give a more complete picture of the child when deciding whether to proceed to diagnostic assessment. | UK |
| 13. | Screening in toddlers and preschoolers at risk for autism spectrum disorder: Evaluating a novel mobile-health screening tool | Kanne, Carpenter et al. 2018 | **Cognoa:**  Two-part classification approach through a mobile app:  1) 15-item parent report, and;  2) 1–2 min home video observation captured via parent smartphone.  A quantitative score attempts to capture the ASD phenotypic severity, which is converted to risk descriptors. | ASD | 18 - 72 months  Participants were separated into 4 age categories for easy comparison (i.e., 18- 30 months, 31- 48 months, 49 - 54  months, and 55- 72months. | To compare Cognoa to the efficacy to: the M-CHAT-R/F; the Social Responsiveness  Scale Second Edition (SRS-2); and the Social Communication Questionnaire (SCQ) and the Child Behaviour Checklist (CBCL) | The experienced clinicians were blind to the Cognoa results, though able to view the results of the other screeners, as these were often part of the clinics’ triage process. | Three  University-based ASD-specialty clinics; | 230 | Unclear -  183 males by author calculation. | - Cognoa performed similarly to other measures, accurately identifying children 71% of the time across the entire 18–72-month age range with an overall higher specificity in detecting ASD compared to the other measures. - Clinicians reported the video component to be the most helpful part of Cognoa. 88% found the summary report helpful, and 76% found that the report and videos alerted them to look for particular behaviours. - Cognoa has a sensitivity of 0.75, and specificity 0.62, with a PPV of 0.83 and NPV of 0.50. (In contrast, the sensitivity of the ASR is 0.76 with a specificity of 0.33, PPV of 0.74, and NPV of 0.35. The CBCL-ASP has a sensitivity of 0.82, and specificity of 0.29, PPV of 0.74, and NPV of 0.40. Cognoa’s specificities are significantly higher when compared to the CBCL-ASP and the ASR (P50.001). Sensitivities were not significantly different. | Majority white (77%) and household income 0-50k (59%). |
| 14. | Developmental screening-Evaluation of an m-Health version of the Parents Evaluation Developmental Status Tools | Maleka, Van Der Linde, et al. 2016 | Smartphone application of the **PEDS tools (**the PEDS and PEDS-DM - see earlier reference) | Developmental delay | 6–18 months 69% (n=142)  19–36  Months 31% (n=65).  M age = 1.94 years; SD = 1.35. | (1) To assess the correspondence between Community health workers (CHWs) trained to use the smartphone applications in the PHC setting as part of their service delivery, and paper-based testing by a speech language pathologist (SLP). | Convenience and disproportionate stratified sampling.  SLP and the CHW blind to each other. | Baby wellness clinics attending a well-baby government PHC facility. | 207 caregivers | 88% mothers | - High positive (100%) and negative correspondence (96%) between the paper based PEDS tools and the smartphone application PEDS tools; and between the SLP and CHW. - Almost perfect (Cohen’s Kappa) inter-rater agreement between conditions (j = 0.873 to j = 0.961). | - South Africa. - 99.9% (n= 206) of children screened were Black. - Sepedi was 44% (n = 90) of the caregivers’ first language, 15% (n = 30) were Tsonga, 12% (n =24) were Zulu speakers, and 29% (n= 63) had other languages as their first language. - Caregivers excluded if not proficient in English. |
| 15. | A Novel System for Supporting Autism Diagnosis Using Home Videos: Iterative Development and Evaluation of System Design | Naznee, et al. 2015 | **NODA smartCapture and NODA Connect.**   - NODA SmartCapture is a mobile phone-based application preinstalled on an iPod touch and a tripod for mounting the iPod that enables parents to easily record clinically relevant prescribed video evidence of their child’s behaviour. It supports recording and uploading 4, up to 10-minute long naturalistic observation diagnostic assessment scenarios, chosen based on pilot research on video-based diagnosis of ASD. Scenarios include: (1) the child playing alone; (2) the child playing with a sibling or peer; (3) a family mealtime; and (4) any behaviour that is of concern to the parent. - The NODA Connect is a Web portal for diagnosticians to direct in-home video collection, access the child’s developmental history, and conduct a remote diagnostic assessment by linking evidence of behaviours tagged in the videos to DSM criteria and making a clinical judgement based diagnostic outcome. - Collaborating diagnostician remotely guided the in-home evidence-collection process by reviewing the videos as they were uploaded and sending alerts to the family as needed to request that they rerecord a particular scenario. | ASD |  | 1. To develop and evaluate the design of an asynchronous system that allows parents to easily collect clinically valid in-home videos of their child’s behaviour and supports diagnosticians in completing diagnostic assessment of ASD.  2. For each family, two diagnosticians, blind to the child’s previous diagnostic status, independently completed an ASD diagnosis via our system. The outcome of the assessment between the 2 diagnosticians, and between each diagnostician was compared to the child’s previous diagnostic status. |  | Unclear how recruited.  In-home | Development stage = 11 clinicians and 6 families;  Evaluation stage = 5 families of children (4 with previous ASD diagnosis and 1 child typically developing) and 3 diagnosticians | ?? | - Without prior training, parents easily (average rating of 4 on a 5-point scale) used the system to record video evidence. - Across all in-home video evidence recorded during field evaluation, 96% (26/27) were judged as clinically useful, for performing an ASD diagnosis. For 4 children (3 with ASD and 1 typically developing), both diagnosticians independently arrived at the correct diagnostic status. In 91% of assessments (10/11) via NODA Connect, diagnosticians confidently (average rating 4.5 on a 5-point scale) concluded a diagnostic outcome that matched with the child’s previous diagnostic status. | USA |
| 16. | Evaluating a web-based clinical decision support system for language disorders screening in a nursery school | Ruiz Duboy, et al. 2014 | The baseline of **Gades KB** was the **Denver Developmental Screening Test**.   - It evaluates four areas of speech and language development: Sensory Reception, Speech Perception, Speech Production, and Pragmatic. - Monthly structured questions, from month 1 to month 72. - The questions belong to two categories: (1) “Alert Milestones” that recommend a visit to the paediatrician within 3 months to re-evaluate the level of language acquisition, and (2) “Alarm Milestones”, that suggest direct referral to a specialist in language disorders. | Language Disorders | 0-6 years | To evaluate a Web-based Clinical Decision Support with the aim to enhance the screening of language disorders. |  | Nursery school | 146 children (94 aged 0 to 3 years; 52 in the 4-6 years stage.  12 educators; 1 language therapist. | 63 boys; 83 girls  56.8%. | - The nursey school SLP accepted Gades’ decisions in 93% of the 0-3 years cases and 67% at the 4-6 years stage. - Therapists disagreed with some questions from Gades KB and suggested adding a few questions about speech production and pragmatic abilities. | Spain |
| 17. | Prima Pietra: a research and clinical program on early  detection and intervention in Italy - | Ruta et al. 2015 | A web-based **M-CHAT** checklist.   - If child failed the checklist, a web- based platform alerted via e-mail the research team and a trained psychologist administered the M-CHAT follow-up over the phone to verify ASD risk. | ASD | 18 months | To assess effectiveness and sustainability of an integrated screening and intervention program for ASD.  **ABSTRACT ONLY** |  | Routine paediatric public health visits. | n = 1146 children screened | Unknown. | N = 76 children failed the MCHAT checklist. At follow-up phone interview n=15 children still at risk of ASD; and 7 out of the 15 children confirmed an ASD diagnosis, with a positive predictive value PPV = 0.47. Presents a good cross-cultural stability. | - Low risk population in Sicily. |
| 18. | Autism Screening with Online  Decision Support by Primary Care  Paediatricians Aided by M-CHAT/F | Sturner et al. 2016 | - **M-CHAT** completed by parents online at home or on a device in the waiting room via a Web system. - Automatically scored and, if positive, the Primary care paediatrician (PCP) completed the follow-up interview. - The flow of the interview was determined by using the failed M-CHAT questions, with the system rescoring after each initially failed item was clarified, and concluding when the initial screening result was either confirmed positive or reversed by the results of the follow-up interview. | ASD | Routine  18- and 24-month visits  M age = 22.9 months.  All but 4 of 98 children in the recommended  age range; these were aged, 14.7, 37, 37, and 40.8 months. | To evaluate the feasibility, validity, and reliability of the M-CHAT/F by PCPs with online prompts at the time of a positive M-CHAT screen.  The PCPs received only brief orientation and access to a  10-minute interactive Web-based demonstration, considerably less than previous studies providing  1.5 days of training in ASD screening.  Specifically, to determine whether PCPs will complete a follow-up interview to a positive M-CHAT during a routine check-up visit and how the results compare with a telephone follow-up interview.  It was reasoned that automated presentation of the exact M-CHAT/F questions for each failed M-CHAT item, and with scoring efficiencies, could facilitate the process. | 5071 children screened with the M-CHAT,  341 (6.7%) were positive.  N = 98 = with complete diagnostic testing and data. | 22 routine health clinics. | 47 PCPs completed 197 M-CHAT/Fs triggered by positive  M-CHAT screens. | As typical with those who screen positive, male 25 (74.5)  Female 73 (25.5). | - The online M-CHAT/F enabled PCPs to clarify positive parent responses to M-CHAT items during well child visits, rather than requiring another visit or call by a trained interviewer. It resulted in significant improvement in PPV compared with the M-CHAT alone. - The computerized algorithm shortened the time of the follow-up interview for children endorsing large number of behaviours because the process stopped automatically when results either confirmed or refuted the initial M-CHAT screen result. | - Maryland, USA. - Practices were mostly (73%) suburban, with some rural (18%) and urban (9%) locations. - Practice estimation of office-level demographic characteristics showed   that 31% of children were Medicaid insured (range, 5%–65%) with 39% white, 33% African American, 16% Asian, and 8% Hispanic.   - Respondents were nearly always mothers (89.8%) and had at least some college education (88.8%). |
| 19. | Early detection of developmental delays in  vulnerable children by community care workers using an mHealth tool | Van der merwe, et al. 2018 | The **PEDS and PEDS: DM** **tools** developed into a smartphone application. | Developmental delay | 1-38 months  (m = 19.2 months; SD = 11.1) | To describe the clinical utility (referral rate, test duration and early detection and perceived value) of a Community Care Worker (CCW)-administered mHealth developmental screening programme. | Exploratory, mixed method | Home-based services  A non-governmental organization that supports families with children who are either infected or affected by HIV/AIDS | 138 children screened | 49% female | Overall referral rate was 69%. Older children (19–38 months) had a significantly higher (p < .05; Chi-Square) referral rate (84%; n = 62) compared to those aged 0–18 months (52%; n = 33).  CCWs perceived mHealth screening as valuable in terms of utility, outcomes and contribution to developmental knowledge for community members and CCWs. | Mamelodi, Gauteng, South Africa.  Mamelodi is one of the largest poverty-stricken  urban populations in the City of Tshwane.  Vulnerable families. Of the families that indicated their monthly income (n=114), 78% (n = 89) received a net income of less than $155 per month. Number of household occupants ranged from 2-10 (4-17%). Most households (76%; n =105) had more than 3 children. |
